# Temporal escalation of Pyrethroid Resistance in the major malaria vector *Anopheles coluzzii* from Sahelo-Sudanian Region of northern Nigeria

**DOI:** 10.1038/s41598-019-43634-4

**Published:** 2019-05-14

**Authors:** Sulaiman S. Ibrahim, Muhammad M. Mukhtar, Jamila A. Datti, Helen Irving, Michael O. Kusimo, Williams Tchapga, Nura Lawal, Fatima I. Sambo, Charles S. Wondji

**Affiliations:** 1 Vector Biology Department, Liverpool School of Tropical Medicine (LSTM), Pembroke Place, L3 5QA United Kingdom; 2 Department of Biochemistry, Bayero University, PMB 3011 Kano, Nigeria; 3 LSTM Research Unit, Centre for Research in Infectious Diseases (CRID), P.O. Box 13591, Yaounde’, Cameroon; 4 Department of Biochemistry and Molecular Biology, Federal University Dutsinma, PMB 5001 Katsina, Nigeria; 5 Department of Biological Sciences, Yusuf Maitama Sule University, PMB 3220 Kano, Nigeria

**Keywords:** Malaria, DNA

## Abstract

Despite the highest global burden of malaria, information on bionomics and insecticide resistance status of malaria vectors is grossly lacking in the densely populated Sahelo-Sudanian region of Nigeria. To support evidence-based vector control we characterised transmission and resistance profiles of *Anopheles coluzzii* populations from three sites in northern Nigeria. High sporozoite infection (~19.51%) was found in the *An*. *coluzzii* populations. A high pyrethroid resistance was observed with only 1% mortality against deltamethrin, a high LD_50_ (96.57 µg/ml), and a high LT_50_ (170.27 min, resistance ratio of ~51 compared with the fully susceptible Ngoussou colony). Moderate carbamate resistance was observed. Synergist bioassays significantly recovered deltamethrin susceptibility implicating CYP450s (mortality = 85%, χ^2^ = 134.04, p < 0.0001) and esterases (mortality = 56%, χ^2^ = 47.31, p < 0.0001). Reduced bed net efficacy was also observed, with mortalities on exposure to the roof of PermaNet3.0 (PBO + deltamethrin) more than 22 times compared to the side panel (deltamethrin). TaqMan genotyping revealed a high frequency of 1014F *kdr* mutation (82%) with significant difference in genotype distribution associated with permethrin resistance [OR = 4.69 (CI:1.53–14.35, χ^2^ = 8.22 p = 0.004]. Sequencing of exons 18–21 of the VGSC led to detection of two additional nonsynonymous mutations, Ile10148Asn and Ser1156Gly. These findings highlight the threats posed by the highly resistant *An*. *coluzzii* to malaria control in Nigeria.

## Introduction

The WHO has set the ambitious goal to reduce global burden of malaria by preventing 90% of malaria-related cases and mortalities by 2030^1^. However, this gradual process is already facing serious setbacks, especially in the WHO African Region which accounts for ~91% of all malaria-related deaths^2^. The recent rebound in malaria transmission with observed increase in cases, as seen in 2016 and 2017^2,3^ is a warning of the risk posed to control and elimination efforts, and suggests that primary regions of interests for pre-elimination needs further attention. Sahelo-Sudanian regions such as northern Nigeria, with high seasonal malaria transmission^4^ are important for monitoring to provide sufficient evidences to support elimination. Nigeria, with the highest burden of malaria, alone account for 27% of global malaria burden; with still increased case incidence between 2010 to 2017^2,3^. Indeed, the Nigerian National Malaria Control Programme (NMCP) Strategic Plan 2014–2020 reported that malaria was responsible for an estimated 20% of under-5 mortality^5^. Elimination efforts will have no chance of succeeding if significant progress is not made in key control areas (like Nigeria) bearing the highest burden of malaria. Unfortunately, control efforts in Nigeria are relatively poor and ill-guided, notably for vector control. Various factors contribute to the failure to make progress in Nigeria. These include a disproportionately low reports of cases through surveillance system (only 8%) and lower coverage of usage for Long-Lasting Insecticidal Nets (LLINs) (60% vs >80% reported for some West African countries, e.g. Senegal, Ghana and Mali)^2,6^. Also, the core malaria intervention tools (e.g. LLINs) are deployed across different eco-epidemiological regions, especially in northern Nigeria without prior knowledge of the composition of the indigenous malaria vector species, let alone their contribution to malaria transmission, their behaviour and insecticide susceptibility status. This lack of information is preventing implementation of evidence-based control strategies. The paucity of information on dominant vector species (DVS) is most extreme in the Sahelo-Sudanian region of the country. With the exception of few focal studies^7,8^ reliable information on the DVS from northern Nigeria is grossly lacking for decades following the comprehensive study conducted in Garki^9^. This is in stark contrast to the situation in southern part of Nigeria (especially the south-west), where significant progress on malaria vector bionomics has been achieved^10–13^.

Over-reliance on the pyrethroids (the only class of insecticides approved by WHO for impregnation of LLINs [http://www.who.int/whopes/en/]) alone has exposed malaria vectors to intense selection with pyrethroid resistance fast becoming the norm across Africa^14^. In the absence of new insecticide to substitute for the pyrethroids it becomes necessary to ensure that the current insecticides used for LLINs remain effective for as long as possible. However, this could only be achieved through management of insecticide resistance and innovative vector control approaches. For example, (i) tailoring control programs to the vector species involved in transmission^15^; (ii) monitoring insecticide resistance in the major malaria vectors to track changes over time and between areas^2^; (iii) establishing molecular markers of insecticide resistance to detect and track its spread to inform control programs^16^ and (iv) evaluating the efficacy of approved major vector control tools.

Prior to this study, in Sahelo-Sudanian regions of Nigeria, contribution of the major malaria vectors to transmission of malaria parasite has not been established comprehensively; though the Sahelo-Sudanian regions (with a high seasonal transmission) offer excellent targets for the malaria elimination through seasonal vector control and chemoprevention^4^. To help boost chances of malaria control in Sudan/Sahel regions and contribute to its elimination agenda, this study provided a comprehensive characterisation of a major malaria vector from three separate localities spanning Sudan and Sahelian regions of northern Nigeria, including species composition, their role in malaria transmission, insecticide resistance profile and the underlying molecular mechanisms driving the resistance in the field.

## Methods

### Study sites and mosquito collection

**Figure 1:**
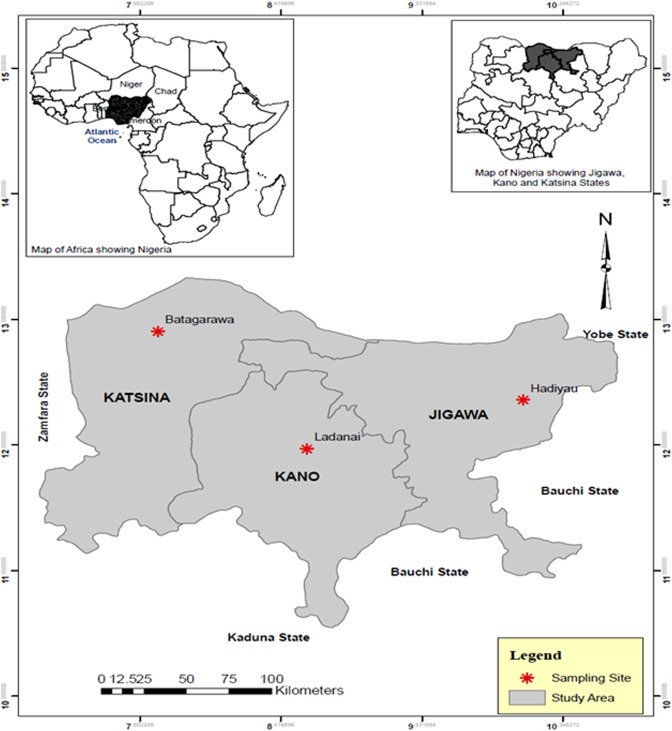
A map showing the three sampling localities in the Sudan/Sahel region of Nigeria.

Blood fed female *Anopheles* mosquitoes resting indoor were collected using Improved Prokopack battery-operated aspirators (John. W. Hock, Florida, USA), from randomly selected houses, in the early morning hours (5:30 am–6:30) in three separate localities in northern Nigeria (Fig. 1): (i) Sahelo-Sudanian Savannah of Hadiyau village (12°21′38″N, 9°59′15″E) in Auyo Local Government, Jigawa State, where rice irrigation is practiced year round; (ii) Ladanai, a locality within the Sudan Savannah of Kano City (11°58′17″N, 8°35′9″E), characterised by a large *Anopheles* breeding site, in running stream used for industrial production of cement blocks; and (iii) Batagarawa town, Batagarawa Local Government in the Sahel Savannah of Katsina State (12°54′17″N, 7°37′11″E). Collections were conducted at Ladanai and Batagarawa for 3 days each, in the first week of the month of August in 2017, coinciding with peak malaria transmission season^4^. For Hadiyau, indoor collection was done for 6 days in the second week of August. The blood fed females were maintained on 10% sugar at 25 °C ± 2 and 70–75% relative humidity for 6 days, until fully gravid. They were then transferred into 1.5 ml tubes individually and forced to lay eggs, as described previously^17^. All F_0_ parents were identified as belonging to *An*. *gambiae* complex using morphological keys^18^ or as *Culex* species. After SINE200-PCR confirmation of the F_0_ parents as *An*. *coluzzii*^19^, DNA from subset of Hadiyau parents were transported to the Liverpool School of Tropical Medicine (LSTM), UK, under the DEFRA license (PATH/125/2012,) for downstream molecular analyses. Egg batches were transferred into paper cups for hatching in insectary at Bayero University Kano, Nigeria. Hatched eggs were pooled into larvae bowls and supplemented with Tetramin^TM^ baby fish food. 2- to 4-days old F_1_ females that emerged were randomly mixed in cages and used for subsequent bioassay experiments.

To establish indoor and outdoor resting densities of the *Anopheles*, infection with *Plasmodium* and source of blood meal, pyrethrum spray collection (PSC) and human landing catches (HLCs) were also conducted during the collection at Hadiyau. Procedures followed protocols as advised by the WHO^20^. PSC was conducted for 1 day in 13 randomly selected houses in early morning hours when occupants in houses were up (6:30 am–7:00 am). Female residents in the selected houses were trained on how to do collection and provided with the materials required. HLC was conducted by 2 adult male participants. Clearance for HLC was obtained from Ethical Sub-committee, Operational Research Advisory Committee, Ministry of Health, Kano, with reference number MOH/off/797/TI/402. Partial collection was done between 7:00 pm–11:00 pm, for 2 days. Collectors were aware of the risk associated with the HLC and were provided with appropriate prophylactic regimen of doxycycline. Mosquitoes were sorted according to morphological keys as either belonging to *An*. *gambiae* complex^18^ or as *Culex* species, and stored individually in PCR strips supplemented with silica gel.

### Species Identification to molecular level

Following morphological identification, genomic DNA was extracted, respectively from 864, 344, and 301 F_0_ female *Anopheles* from Hadiyau, Ladanai and Batagarawa, which laid eggs successfully. DNA extraction was performed following the protocol of LIVAK^21^. Species identification to the molecular level was carried out using the SINE200 PCR^19^. For all *An*. *gambiae s*.*l*. collected from PSC and HLC PCR identification was also carried out as explained above after morphological identifications.

### Estimation of entomological and parasitological parameters of transmission

#### Indoor resting densities and outdoor host-seeking behaviours

The *Anopheles* indoor resting density (IRD) was calculated from the number of *Anopheles* caught using the PSC relative to the number of houses sampled, following the procedures outlined by WHO^20,22^. For the outdoor feeding behaviour man landing frequency (MLF) was calculated from the number of *Anopheles* captured while landing on the 2 volunteers in the 2 days of collection^22^.

#### Human blood index and biting rate

To establish anthrophophilic index, blood-fed female *An*. *gambiae s*.*l*. collected from Hadiyau, using PSC were dissected <48 hours after collection to separate heads and thoraces from the abdomens. 143 blood fed females were dissected, heads/thoraces and abdomens prepared for DNA extraction as outlined in WHO guidelines^20^. DNA was extracted from these head/thoraces and abdomens using the DNeasy Blood and Tissue Kit (QIAGEN, Hilden, Germany) according to manufacturer’s protocol. SINE200 PCR protocol was utilised first to establish the species identity. The human blood index was established from the proportion of blood fed *Anopheles* that have fed on humans relative to the total number of blood fed female *Anopheles* caught, following a cocktail PCR of Kent and Norris *et al*.^23^. Human biting rate was also estimated from the number of human blood fed mosquitoes relative to the total number of occupants in the houses.

#### Estimation of sporozoites infection rate and entomological inoculation rate

93 blood fed female *An*. *coluzzii* collected indoor using PSC, and 37 females which laid eggs (collected with aspirators) were used to detect sporozoite with a TaqMan genotyping approach previously described^24^. Real-time PCR MX 3005 (Agilent, Santa Clara, USA) was used for the amplification. 1 μl of gDNA extracted from each female head/thorax was used as a template for PCR, with an initial denaturation at 95 °C for 10 min, followed by 40 cycles each of 15 sec at 95 °C and 1 min at 60 °C. Primers described by Bass^24^ were used together with two probes labelled with fluorophores, FAM to detect *Plasmodium falciparum*, and HEX to detect combination of *P*. *ovale*, *P*. *vivax* and *P*. *malariae*. Positive controls (known FAM+ and OVM+) were used in addition to a negative control in which 1 μl of dH_2_0 was added. To validate findings of the TaqMan assay a nested PCR of Snounou and colleagues^25^ was carried out using all the TaqMan-positive samples. Sporozoite rate was calculated as percentage of mosquitoes with sporozoites relative to the total number of the females examined^20^ and entomological inoculation rate was estimated from the sporozoite rate and human-biting rate, as previously described^26^.

### WHO insecticide susceptibility bioassays

Insecticide susceptibility bioassays were performed following the WHO protocol^27^ with the insecticides from four major public health classes. At least four replicates of 20–25 adult F_1_ female *Anopheles* per tube were used for each insecticide, alongside 25 unexposed females (control). To confirm the efficacy of the papers, the fully susceptible *An*. *coluzzii* (Ngoussou strain)^28^ was tested first or simultaneously with the experimental populations. For Hadiyau, 11 insecticides were tested, including: (i) the type I pyrethroid: permethrin (0.75%); (ii) the type II pyrethroids: deltamethrin (0.05%), λ-cyhalothrin (0.05%), α-cypermethrin (0.05%) and cyfluthrin (0.15%); (iii) the organochlorine: DDT (4%); (iv) the carbamates: bendiocarb (0.1%) and propoxur (0.1%); and (v) the organophosphates: malathion (5%), pirimiphos-methyl (0.25%), chlorpyrifos-methyl (0.4%) and fenitrothion (1%, with 2 hr exposure^29^). For Ladanai populations, permethrin, deltamethrin, DDT, bendiocarb and malathion were tested; while for Batagarawa populations, permethrin, deltamethrin, DDT, bendiocarb and pirimiphos-methyl were tested. Availability of F_1_ females raised to adulthood successfully accounted for the differences seen in the number of insecticides tested for the different sites. In all the three populations knockdown rates were recorded for permethrin, deltamethrin and DDT during the exposure, in intervals of 15 min, 30 min, 45 min and 1 hr. After 1 hr exposure mosquitoes were transferred to holding tubes and supplied with 10% sugar. Mortality was scored 24 hr after exposure. Populations were considered susceptible to an insecticide where mortality was >98%, suspected to be resistant (moderately resistant) where mortality is between 90–98%, and resistant where mortality was found to be <90%^27^.

### Synergist bioassays

To establish the potential insecticide detoxification enzyme systems responsible for resistance synergist bioassays were conducted with 2–5 days old F_1_ females from Hadiyau with 4% piperonyl butoxide (PBO: an inhibitor of CYP450s and carboxylesterases^30^), and with 0.25% S,S,S-tributylphosphorotrithioate (DEF: an inhibitor of carboxylesterases)^31^ against 0.05% deltamethrin. In addition, potential role of glutathione S-transferases (GSTs) in DDT resistance was also investigated by pre-exposure to 8% diethyl maleate (DEM). About 20–25 females were initially pre-exposed to a synergist for 1 hr and then transferred to tubes containing deltamethrin or DDT, for 1 hr^27^. Mosquitoes were treated as in conventional bioassays described above and mortalities scored after 24 hr. Two controls were set up: (i) 25 females exposed to only control paper with neither synergist, nor any insecticide; and (ii) 25 females exposed to PBO only.

### Efficacy of insecticide-treated nets using Cone bioassays

The efficacy of PermaNet 3.0 (a PBO-deltamethrin combination bed net) supplied by Vestergaard (https://www.vestergaard.com/permanet-3-0) was tested with Hadiyau females using the WHO cone bioassay protocol^32^ with modification in the number of mosquitoes per replicate. Five different sections of the net, each measuring 30 × 30 cm were cut separately for both the roof (PBO + deltamethrin) and the side (deltamethrin only) of the PermNet 3.0. Five females were introduced into each of the 4 cones fixed onto the piece of net, making a total of 20 females per experiment. This was then replicated 5 times exposing the mosquitoes in each round for only 3 min. The females were then transferred to holding paper cups, supplied with sugar and allowed to rest for 24 hr before mortality was scored. Controls comprise 8 replicates each of 5 females in two pieces of untreated nets. In addition, 8 replicates each of 5 females of the lab susceptible Ngoussou colony were also used as positive control.

### Estimation of resistance intensity with time-course and dose-response bioassays

To establish the strength of pyrethroid resistance with time, additional bioassays were performed with 0.05% deltamethrin, using the Hadiyau population. Deltamethrin was chosen due to its wide use in pyrethroid bed nets, and because the Hadiyau populations exhibited the highest resistance to it. Cohorts of 20–25 F_1_ females in 4 replicates were exposed in time-course bioassays for 60, 120, 180, 240 and 300 min to establish the time required to kill 50% of the females (LT_50_). Protocols were as described above in conventional bioassays, except for variation in time. The fully susceptible Ngoussou colony were also exposed to discriminating concentration of deltamethrin in time-course bioassays spanning 2.5, 5, 10, 15, 30, 45 and 60 min, and LT_50_ calculated. Resistance intensity was established by comparing the calculated LT_50_ from the Hadiyau populations to that of the Ngoussou.

The diagnostic dose which kills 50% (LD_50_) of the F_1_ females from Hadiyau was also established using the CDC bottle bioassays. This was conducted with ranges of deltamethrin concentration starting from 200 µg/ml, serially diluted 2-fold into 100, 50, 25, 12.5 (the discriminating dose in the conventional CDC protocol^33^), and 6.255 µg/ml, respectively. 4 replicates of 20–25 F_1_ females were used for each concentration. Mosquitoes were exposed for 1 hr, then transferred into holding cups and supplied with 10% sucrose. Mortality was recorded at 24 hr.

### Polymorphism analysis of the voltage-gated sodium channel

#### Genotyping of L1014F and L1014S kdr mutations

To assess the frequency of the *kdr* mutations in the Hadiyau *An*. *coluzzii*, DNA from 31 blood fed F_0_ females, indoor-collected and which laid eggs successfully were randomly selected and screened with TaqMan real-time PCR assay (using Agilent Mx3005 qRT-PCR thermocycler). These females were genotyped for the presence of 1014F and 1014S *kdr* mutations following the protocols established by Bass and colleagues^34,35^. In a total volume of 10 µl comprise of 5 µl Sensimix (Bioline), 0.25 µl of 40x Probe Mix coupled to allelic-specific primers, 4.25 µl of dH_2_0, and 1 µl of genomic DNA was added. Thermocycling conditions were initial 10 min at 95 °C, followed by 40 cycles each of 92 °C for 15 sec, and 60 °C for 1 min. Two probes labelled with fluorochromes FAM and HEX were utilised to detect the mutant alleles and the wild type susceptible alleles, respectively. Genotypes were scored from scatter plots of results produced by the Mx3005 v4.10 software. Three positive samples of known genotypes: (i) homozygote resistant for 1014F or 1014S *kdr*, (ii) heterozygote for 1014F or 1014S *kdr*, and (iii) susceptible L1014 were added as positive controls for each of the two experiments to determine the *kdr*-West or *kdr*-East, respectively. 1 µl of dH_2_0 was added to the negative control well.

To assess the relation between the *kdr* genotypes and pyrethroids resistance phenotypes, 92 permethrin resistant individuals and 18 susceptible individuals were genotyped for the presence of 1014F and 1014S *kdr* mutations. Protocol was as explained above. However, with no mortalities obtained from conventional bioassays with deltamethrin and with only 7 individuals dead from exposure with permethrin, bioassays were repeated with permethrin to get a minimum of 16 susceptible females. Correlation between the L1014F *kdr* genotypes and permethrin resistance phenotype was assessed by estimating the odds ratio (OR) and the statistical significance based on Fisher exact probability test.

#### Sequencing of exons 18–21 of the voltage-gated sodium channel for novel kdr mutations

To detect potential mutations in the para-type sodium channel, a fragment of exons 18–21 (harbouring the 1014 *kdr* mutation in exon 20 of resistant populations) from the Hadiyau population and the Ngoussou strain were amplified and sequenced. Briefly, RNA was extracted from 5 batches each of 10 deltamethrin-resistant Hadiyau mosquitoes and from Ngoussou strain using the PicoPure RNA isolation Kit (Arcturus, Applied Biosystems, USA). cDNA was synthesized from extracted RNA using SuperScript III (Invitrogen, USA) with oligo-dT20 and RNAse H (New England Biolabs, USA). Amplification was carried out using the following primer pair COLZkdrEx18F: ATAATGTGGATAGATTCCCCG, and COLZkdrEx21R: CTTCGTCAATTCCTAGATCT. To 12.5 μl of the 2x AccuStartII PCR SuperMix (QuantaBio, Beverly, Massachusetts) containing optimised concentrations of MgCl_2_ and dNTP mixes, 0.2 μM each of the forward and reverse primer was added, followed by 1 μl cDNA. 10.5 μl of dH_2_0 was added to obtain a total volume of 25 μl. Amplification was carried out using the following conditions: initial denaturation of one cycle at 94 °C for 3 min; followed by 35 cycles each of 94 °C for 30 sec (denaturation), 60 °C for 30 s (annealing), and extension at 72 °C for 1 min; and one cycle at 72 °C for 5 min (final elongation). PCR products (~750 bp) were cleaned individually with QIAquick^®^ PCR Purification Kit (QIAGEN, Hilden, Germany) and cloned into pJET1.2/blunt according to manufacturer’s protocol (ThermoFisher Scientific, MA, USA). These were then used to transform the *Escherichia coli* DH5α, plasmids miniprepped with the QIAprep^®^ Spin Miniprep Kit (QIAGEN, Hilden, Germany) and sequenced on both strands using the pJET1.2 sequencing primers.

Polymorphisms were detected through manual examination of sequence traces using BioEdit version 7.2.3.0^36^ and CLC sequence viewer (QIAGEN, Hilden, Germany). Sequences have been deposited in GenBank for accession numbers.

### Statistical analyses

R version 3.5.0 (https://cran.r-project.org/bin/windows/base/) was utilized for statistical analyses to calculate Odds Ratio (epiR package) for the 1014F *kdr* genotyping, and for the estimation of LT_50_ and LD_50_ probit analyses (glm with MASS package). Result of mortalities from synergist-insecticide exposure and temporal variation in pyrethroid resistance were compared with those obtained from exposure to synergists alone using a two-tailed Chi-Square test of independence as implemented in GraphPad Prism 7.02 (GraphPad Inc., La Jolla, CA, USA).

## Results

### Mosquito species distribution and composition

All the F_0_ female *Anopheles* collected indoor using aspirators and PSC, as well as outdoor from HLC were *An*. *coluzzii*. For all the 3 sites, 4,276 mosquitoes were caught indoor. These comprise 3,491 *Anopheles*: 2,661 blood fed females, 637 unfed and 193 males; and 785 Culex. Of the *Culex*, 568 were blood fed females, 127 unfed, and 90 were males (Supplementary Fig. S1b). 2,263 of all the *An*. *coluzzii* were collected from Hadiyau, with ~50% of them (1,736) blood-fed (Supplementary Fig. S1a). In contrast, 226 *Culex* mosquitoes were caught from Hadiyau, 131 blood-fed females and 52 unfed females (Supplementary Fig. S1b). For Ladanai, *An*. *coluzzii* constitutes 18% of the total mosquitoes caught from all 3 sites, with 508 blood-fed and 107 unfed (Supplementary Fig. S1a). Ladanai *Culex* constitutes 17% of the total *Culex* species caught from all sites, with 88 blood-fed females and 21 unfed (Supplementary Fig. S1b). The greatest proportion of *Culex* mosquitoes were caught at Batagarawa (424 in total), 349 of them blood-fed and 54 unfed (Supplementary Fig. S1b). 559 *An*. *coluzzii* were caught at Batagarawa, of which 417 were blood-fed and 114 were unfed.

A total of 225 mosquitoes were caught from PSC, 196 of them *An*. *coluzzii* (143 blood-fed females, 38 unfed, and 15 males), while 29 *Culex* were also recovered (18 blood-fed females, 7 unfed females and 4 males). Of the 113 female mosquitoes captured with the HLC 96 were *An*. *coluzzii* and 17 of them *Culex*.

### Entomological and parasitological parameters of transmission

#### Indoor and outdoor resting densities

Indoor resting density of Hadiyau *An*. *coluzzii* was calculated from the 181 females collected in 8 houses as 22.625 female *Anopheles* per room. For outdoor, the 2 collectors caught 52 and 44 female *Anopheles* respectively, in two days. The man-landing frequency (MLF) of the *An*. *coluzzii* females was estimated as 24 (CI: 20–28).

#### Human blood index and biting rate (Anthropophilic index)

DNA extracted from 143 blood fed females collected using PSC was analysed for blood source. 139 of the abdomens contained human blood, 3 abdomens contained cow blood and only one of abdomen contained blood from a goat. The human blood index was found to be very high; 97.2% of the foraging female *An*. *coluzzii* have fed on humans. The human biting rate was calculated as ~2.05 (CI: 0.88–3.52) bites per person per night.

#### Estimation of sporozoites infection rate and entomological inoculation rate (EIR)

A total of 130 DNA-extracted heads/thoraces (93 from PSC-caught *An*. *coluzzii*, and 37 from females that have laid eggs) were used to establish infection with *Plasmodium*. 25 heads/thoraces were found to be infected with *P*. *falciparum* (F+ = 25), corresponding to a sporozoite rate of 19.51 (CI: 9.76–29.25). One additional sample tested positive for *P*. *vivax/malariae/ovale*. The EIR was calculated from the *P*. *falciparum* sporozoite rate and human biting rate as 0.39 ± 0.21 infective bites per person per night. Validation of the TaqMan results using nested PCR^25^ confirmed infection in 25 samples, including the non-*falciparum* sample which turn out to be *P*. *vivax*.

### WHO insecticide susceptibility bioassays

2,084 F_1_ females were utilised for the conventional WHO bioassays: 1,136 from Hadiyau, 486 from Batagarawa, and 462 from Ladanai. Overall, knockdown was very low for pyrethroids and DDT, and increased with exposure time. However, mosquitoes were least knocked down by DDT, followed by deltamethrin, compared to permethrin (Supplementary Fig. S2). Hadiyau populations exhibited the lowest, but not significantly different knockdown with permethrin, with effect at 1 hr of exposure one-third the values obtained for the Batagarawa and Ladanai populations (χ^2^ = 2.59, df = 1, p = 0.10, compared to Batagarawa; and χ^2^ = 3.06, df = 1, p = 0.08 compared to Ladanai). An exception in the trend was observed with cyfluthrin tested against Hadiyau populations only (Supplementary Table S1). Exposure to this type II pyrethroid inflicted increased knockdown, reaching ~30% at 45 min, and ~40% at 1 hr. These differences were statistically significant compared to the other two type II pyrethroids, deltamethrin (χ^2^ = 31.8, df = 1, p = 1.73 × 10^−8^) and λ-cyhalothrin (χ^2^ = 30.1, df = 1, p = 0.001) tested with the Hadiyau population.

**Figure 2:**
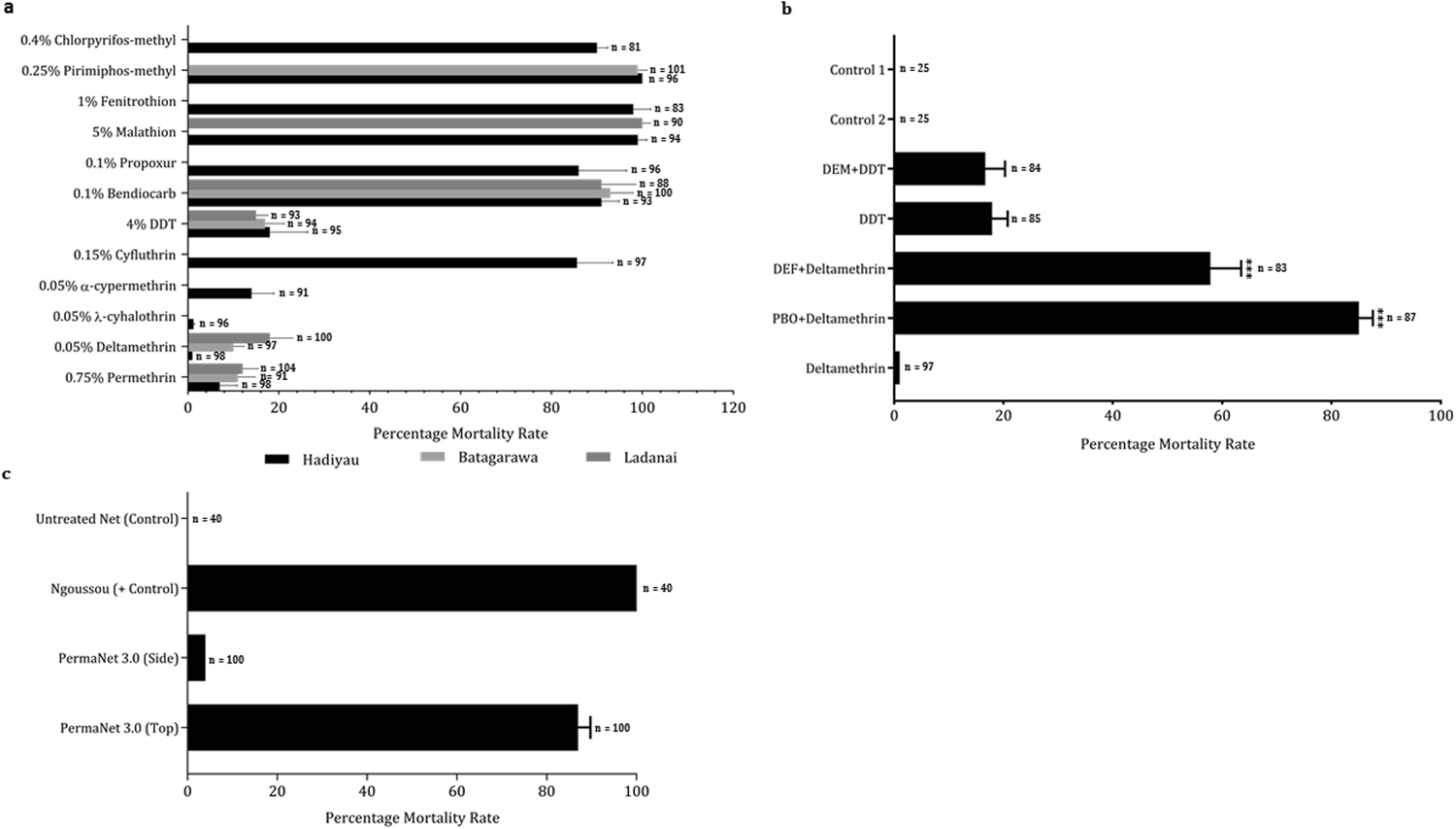
Resistance profiles of F_1_ *An*. *coluzzii* females. (**a**) Results of WHO bioassays with insecticides from different classes. Results are average of percentage mortalities from 4 replicates each ± SEM. (**b**) Effect of pre-exposure to synergists PBO and DEF against deltamethrin, and with DEM against DDT. Results are average of percentage mortalities from 4 replicates each ± SEM. Control 1: mosquitoes that were neither exposed to any synergist nor any insecticide; Control 2: mosquitoes exposed to PBO only. ***=statistically significant at p < 0.0001 from two tailed χ^2^ square test of independence, between results from synergists bioassay and conventional bioassays with deltamethrin. (**c**) Results of cone bioassays with PermaNet 3.0. Ngoussou (+control) exposed to the side panels of PermaNet 3.0 only. Results are average of percentage mortalities ± SEM.

The fully susceptible Ngoussou strain exhibited 100% mortalities against all insecticides tested. Field *An*. *coluzzii* populations were highly resistant to type I and type II pyrethroids, except for cyfluthrin which was tested only with Hadiyau populations (Fig. 2a). For permethrin, lowest mortality was observed with Hadiyau populations, at 7% (95%, CI: 4–11), compared to 11% (CI: 7–15) and 12% (CI: 8–16) observed with Batagrawa and Ladanai populations. Lowest mortality was obtained with Hadiyau populations exposed to deltamethrin (1%, CI: 0–1), in contrast to 10% (CI: 8–13) and 18% (CI: 13–23) obtained with Batagarawa and Ladanai populations, respectively. Lowest resistance was also recorded in Hadiyau populations exposed to type II pyrethroids λ-cyhalothrin (1%, CI: 0–1) and α-cypermethrin (14%, CI: 9–19). Cyfluthrin exhibited the highest toxicity of all pyrethroids tested, with a mortality of 86% (CI: 78–93) in Hadiyau populations. For all the three sites, level of mortality from DDT exposure was comparable, on average less than 20%.

In all the three sites mosquito populations were more sensitive to carbamate and organophosphate insecticides compared to pyrethroids and DDT. For example, in all three sites reduced mortalities were observed towards bendiocarb, ranging from 91% in Hadiyau (CI: 87–95) and Ladanai (CI: 84–98), to 93% in Batagarawa (CI: 88–98) (Fig. 2a). Hadiyau populations exhibited moderate mortalities to propoxur, another carbamate (86% mortality, CI: 75–96). Except for chlorpyrifos-methyl [moderate resistance in Hadiyau populations (90% mortality, CI 88–92)], full susceptibility was observed with the organophosphate insecticides, with mortalities in range of 98% (CI: 94–102) with fenitrothion for Hadiyau populations, to 99–100% for malathion and pirimiphos-methyl, in all the three respective populations tested (Fig. 2a).

### Estimation of intensity of resistance

To establish levels of resistance WHO tube bioassays were conducted with deltamethrin varying exposure times from 60 min to 300 min. The LT_50_ of Hadiyau *An*. *coluzzii* populations was estimated as 170.27 min [95% CI: 159.59–180.96, Fiducial] (Supplementary Fig. S3a). The LT_50_ for the Ngoussou colony was estimated as 3.320 min [CI: 2.67–3.97] (Supplementary Fig. S3b). The resistance ratio between Hadiyau populations and the Ngoussou was calculated as 51.28.

Strength of pyrethroid resistance was also estimated by varying the concentrations of deltamethrin from 6.25 µg/ml through to 200 µg/ml in a CDC bottle bioassay (Supplementary Fig. S3c). LD_50_ was very high, estimated as 96.57 µg/ml [95% CI: 85.59–107.55, Fiducial].

### Investigating the potential role of metabolic resistance using synergist bioassays

To investigate the possible role of metabolic enzyme systems in the observed resistance, bioassays were conducted with three synergists PBO and DEF against deltamethrin, as well as DEM against DDT. A very high recovery of susceptibility was obtained from Hadiyau populations from pre-exposure to PBO and DEF against deltamethrin, with mortalities on average 85 times and 56 times the values obtained in conventional bioassays without the synergists (Fig. 2b). Two-tailed test of independence indicated that the associations between the mortality and PBO pre-exposure are highly significant (χ^2^ = 134.104, df = 1, p < 0.0001). The same statistical significance was also obtained with pre-exposure to DEF (χ^2^ = 47.31, df = 1, p < 0.0001). These support the possible role of P450 monooxygenases and esterases in the observed deltamethrin resistance. No recovery of mortalities was seen on pre-exposure to DEM followed by DDT. No mortality was observed with control 1 (exposed to neither synergists nor insecticides) and control 2 (exposed to PBO only).

### Determination of insecticide-treated bed nets efficacy

To evaluate the efficacy of conventional and combination long-lasting bed nets, cone bioassays were conducted. Initially, both the roof and side of the PermaNet 3.0 were used against the fully susceptible Ngoussou *An*. *coluzzii*, with 100% mortality after 24 hr. Hadiyau populations were then tested with the net. A low efficacy was observed with the side of the net (impregnated with deltamethrin only) with an average knockdown of only 3% ± 0.4, and average mortality of only 4% ± 0.88 in 24 hr (Fig. 2c). In contrast, exposure to the roof of the net (deltamethrin + PBO) resulted in significant recovery of susceptibility with average knockdown of 59% ± 4.8 and a 24 hr mortality of 87% ± 2.88. This mortality is on average 22-fold compared to the observation with the side of the net (χ^2^ = 133.9, df = 1, p < 0.0001).

### Increased pyrethroid and DDT resistance Intensity in *An*. *coluzzii* populations

To determine the temporal changes in resistance levels in Hadiyau populations data published in 2014^7^ (from collections done in 2009) and the results from this study were compared. Overall, increased pyrethroids and DDT resistance was observed. For example, in our study from 2014^7^ we have established a percentage knockdown of about 30% respectively, for the type II pyrethroids λ-cyhalothrin and deltamethrin; and mortalities of 52% and 78%. In less than a decade the resistance has escalated with percentage knockdown of less than 5% in Hadiyau populations in 2017 and mortalities of about 1% for both λ-cyhalothrin (χ^2^ = 65.562, df = 1, p = 5.38 × 10^−16^) and deltamethrin (χ^2^ = 120.24, df = 1, p = 5.59 × 10^−18^ for deltamethrin) compared to 2009 populations. The DDT resistance has more than double, with mortalities of only 18% compared to 44.6% we have reported previously (χ^2^ = 15.586, df = 1, p = 0.0079). The only exception was the organophosphate malathion of which 99–100% mortalities were recorded between 2011–2017.

### Polymorphism analysis of the voltage-gated sodium channel

#### Investigation of the role of 1014F and 1014S kdr mutations on pyrethroid resistance

**Figure 3:**
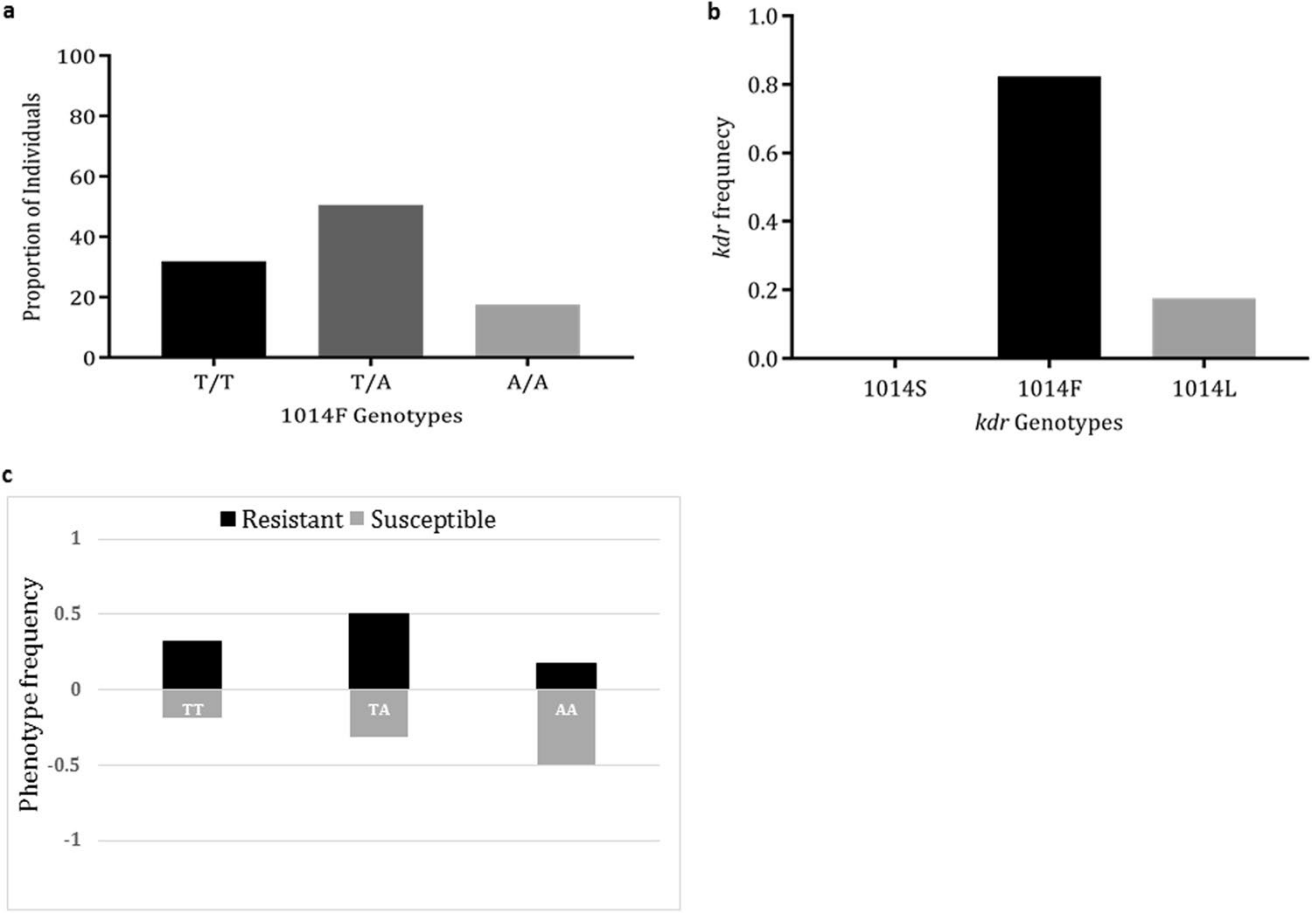
Polymorphism analyses of the Hadiyau *An*. *coluzzii* voltage-gated sodium channel. (**a**) Proportion of female *An*. *coluzzii* with 1014F *kdr* genotype; (**b**) the *kdr* frequency in female wild population. (**c**) Correlation between the L1014F *kdr* genotypes and permethrin resistance phenotype. The frequency of each genotype is plotted in each phenotype to indicate differences in survival between the genotypes (T/T: resistant kdr genotype; A/T: heterozygote; A/A: wild type susceptible). (**d)** Phylogenetic tree of the exons 18–21 fragment of Hadiyau populations vs Ngoussou cohort.

To establish the frequency of *kdr* mutations in the field, 30 Hadiyau F_0_ females that laid eggs were randomly selected and genotyped for the L1014F and L104S *kdr* mutations. A high frequency of 1014F mutations were observed with 10 of the females (33.33%) scored as homozygote resistant (T/T), 15 as heterozygote (50% T/A), and only 5 (16.66%) females were susceptible (A/A) (Fig. 3a). The 1014F *kdr* genotype frequency was estimated as 0.83 in the wild populations (Fig. 3b). In contrast, no L1014S *kdr* mutation was observed in the field.

Additionally, 91 permethrin-resistant and 16 dead F_1_ females were successfully genotyped for 1014F and 1014S *kdr* mutations. As in the field high frequency of 1014F *kdr* mutation was obtained, with 29 females homozygote resistant (T/T), and 46 heterozygote (T/A) for the 1014F *kdr* mutation (Fig. 3c, Table 1). Only 16 of the resistant females were found to be homozygote susceptible (A/A). The 1014F *kdr* genotype frequency was established as ~0.82. The 1014S *kdr* mutation was not detected in these mosquitoes, as well. A significant correlation was observed between permethrin resistance and presence of 1014F mutation [Odds Ratio of 4.69 (95% CI: 1.53–14.35, χ^2^ = 8.219, p = 0.004)] (Table 1) when comparing the frequency of the resistance with susceptible allele in all alive and dead females. Similar association was observed in the RR females in relation to the SS females [OR of 4.83 (CI: 1.12–20.82, χ^2^ = 4.97, p = 0.026)] compared to the association observed between the latter (SS) and the heterozygote females (RS) [OR of 4.6 (CI: 1.31–16.12, χ^2^ = 6.31, p = 0.012)].

**Table 1:** Correlation between the 1014F allele frequency and the permethrin resistance phenotype in Hadiyau populations.

Population	Phenotype	n	L1014F Alleles			% *kdr* frequency (RR + RS)	*kdr* allele	Odds Ratio (OR)	χ^2^ (p value)
			TTT (RR)	TTT/A (RS)	TTA (SS)				
Hadiyau ♀	Alive	91	29 (31.86%)	46 (50.54%)	16 (17.58%)	75 (82.41%)	0.83	4.69 (1.53–14.35)	8.22 (0.004)
	Dead	16	3 (18.75%)	5 (31.25%)	8 (50%)	8 (50%)	0.50		
Total		107	32 (29.90%)	51 (47.66%)	24 (22.42%)	83 (78.50%)			

n = number of successfully genotyped individuals. Numbers in brackets represent percentage frequency. TTT: homozygote resistant alleles (RR); TTT/A: heterozygote resistant; and TTA: homozygote susceptible.

#### Sequencing of the Exons 18–21 fragments of the voltage-gated sodium channel for kdr mutations

**Figure 4:**
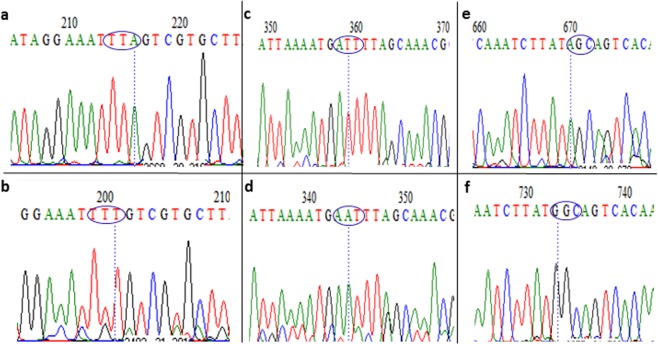
Analysis of the polymorphism of a fragment of the voltage-gated sodium channel (VGSC) gene spanning the L1014F/S mutation. Polymorphic codons for (**a**,**b**). Ngoussou vs Hadiyau, 1014 position. (**c**,**d**) Ngoussou vs Hadiyau, 1048 position; and (**e**,**f**): Ngoussou vs Hadiyau, 1156 position, are in blue circle.

A 747 bp fragment of the VGSC spanning exons 18–21 and encompassing the 1014 codon was amplified and sequenced. In addition to confirmation of the 1014F replacement (Fig. 4a,b) in the Hadiyau populations with respect to Ngoussou (TTA- > TTT), two additional non-synonymous mutations were discovered (Fig. 4c–f, Supplementary Fig. S4): (i) ATT in Ngoussou coding for isoleucine (Ile^1048^), replaced with AAT coding for asparagine (Asn^1048^) in Hadiyau populations; (ii) AGC in Ngoussou coding for Ser^1156^, replaced with GGC for Gly^1156^ in Hadiyau populations. However, the potential role of these mutations in pyrethroids resistance need further validation.

## Discussion

The Sahel region characterised with high seasonal transmission offers excellent opportunity for control and elimination of malaria. Unfortunately, control efforts in these regions, especially Nigeria are very poor, partly due to lack of information on the bionomics and resistance profile of the dominant vector specie(s). To facilitate evidence-based control measures and resistance management we identified the major malaria vector in the Sahel/Sudan region of Nigeria, established its role in *Plasmodium* transmission, its resistance profile, and the possible underlying molecular mechanisms driving the resistance in the field.

The fact that *An*. *coluzzii* (the M molecular form of *An*. *gambiae*) was the only *Anopheles* species collected in the three separate sites in northern Nigeria, and within the rainy season (coinciding with the peak in malaria transmission) suggests that these species is the major malaria vector in this Sahelo-Sudanian region. Indeed, studies carried out decades ago have described *An*. *gambiae s*.*l*. as the major malaria vector in northern Nigeria^37,38^. Also, contrary to the previous observations that *An*. *arabiensis* tend to predominate in arid savannas whereas *An*. *gambiae* is the dominant species in humid forest zones^39–41^, the findings of *An*. *coluzzii* as the major malaria vector in two localities between 2009 and 2011 in northern Nigeria^7^ and the confirmation of its dominance in three separate sites in this study suggests that this malaria vector has probably adapted well in drier Sahel/Sudan region of Nigeria. In a study carried out less than 2 decades ago Awolola and colleagues^42^ have described *An*. *gambiae* s.s. (the S molecular form sibling species of *An*. *gambiae*) as the major malaria vector in the savannah.

Because information on malaria transmission indices in the Sudan/Sahel savannah of northern Nigeria is scanty it becomes necessary to compare our findings with studies carried 4–5 decades ago. The indoor resting density we have calculated for Hadiyau is higher in comparison to the findings of Garki project (IRD of 4.1–16)^9^. The human biting rate per person per night was however, lower than the range of man-biting rate reported in the Garki Project. The sporozoite rate we have founded was roughly 3 times higher than the rates White and Rosen (SR: 5.5% to 9.3%)^43^ have described in *An*. *gambiae* populations from Kaduna (a Sudan savannah of northern Nigeria). The Garki Project has described lower sporozoite rate of no more than 3% as well, and the entomological inoculation rate we have calculated was within the ranges described in the above project. Sporozoite rate of 11% has been described in *An*. *gambiae s*.*l*. populations from rainforest/swampy regions of south-eastern Nigeria^44^. Overall, the *An*. *coluzzii* populations from Hadiyau were highly endophilic and anthropophilic and pose a great risk as they have a very high infection rate.

Comparison of our findings with previous works suggests that insecticide resistance in northern Nigerian *An*. *coluzzii* has escalated in a few years. We have previously reported in 2014 *An*. *coluzzii* populations from two sites in the Sudan savanna of northern Nigeria exhibiting mortalities with pyrethroids in the range of between 52–78%^7^. Also, studies carried out in 2014 in northeast and south-west Nigeria^8,12^ has reported a high mortality with pyrethroids, and full susceptibility to carbamates and organophosphates. Our findings in this study suggest a dramatic rise in pyrethroid resistance in less than a decade; for all three sites mortalities for all pyrethroids, except for cyfluthrin were less than 20%, a four-fold decrease in mortality on average. In less than a decade these mortalities have become reduced to only 1% for the type II pyrethroids. Also, unlike previous studies, for the first time we have found resistance, though moderate to carbamate in the field populations from northern Nigeria, as well as possible resistance to the organophosphate, chlorpyrifos-methyl. The extremely high pyrethroid resistance observed in the Hadiyau populations is evident in its high LT_50_ with deltamethrin which is on average more than double the values described previously for the Tororo (Uganda) and Tiefora (Burkina Faso) resistant populations *of An*. *gambiae*, respectively with permethrin^45^. The LD_50_ we obtained with deltamethrin was also on the high side compared to data available from previous studies; it was on average three times the ranges described for the highly resistant VK7 *An*. *gambiae* populations from Burkina Faso^46^.

The high recovery of resistance when mosquitoes were pre-exposed to PBO and DEF indicates the potential role of P450 monoxygenases and carboxylesterases, respectively. Synergists are increasingly utilised for initial investigation of contribution of metabolic resistance in *An*. *gambiae* populations^47,48^. The results obtained with the synergists agrees with the outcome of the cone bioassay with low mortalities obtained with deltamethrin-impregnated side panels of the PermaNet 3.0 increasing 15-fold when compared with the top panel of the net. Differences in mortalities between the two sides of this LLIN has been recently reported in *An*. *gambiae s*.*l*.^49^ and *An*. *funestus*^50^. However, a single net utilised for the cone bioassays is one of the shortcomings of this study. Future work should assess this efficacy with several independent batches of nets.

The high frequency of 1014F *kdr* mutation observed in the Hadiyau population was similar to the frequency we have reported in *An*. *coluzzii* collected from same locality, in 2009^7^. The absence of the 1014S *kdr* mutation in the Hadiyau population is however, consistent with the absence of *An*. *arabiensis* at Hadiyau, since this mutation was only found previously in a single female *An*. *arabiensis* in the same locality. In addition, the findings of two more mutations in the IIS6 domain indicates possible selection of additional knockdown phenotype that should be investigated thoroughly. However, future studies on these populations should also assess the additional role that other mutations such as N1575Y could play in this resistance profile. Indeed, as it has been shown that N1575Y mutation confers additional resistance to insecticides^51^, and that the N1575Y-L1014F double mutant channel was more resistant to pyrethroids than the L1014F mutant one^52^.

## Conclusion

In less than a decade, pyrethroid resistance in *An*. *coluzzii* from northern Nigeria has escalated, posing a serious setback to the effort to reduce malaria burden by 90%, in line with WHO projection by the year 2030. Of considerable alarm also is the carbamate resistance on the rise in this field populations and the suspected resistance to one organophosphate, which would confound control measures-the indoor residual spraying. It is of utmost importance to continue surveillance of this resistance and its underlying mechanisms in these areas to inform the malaria control program on its progress. This will help in implementation of effective evidence-based control measures.

## Supplementary information

Supplementary Information

## Data Availability

The nucleotide sequences generated in this study are available in the GenBank (accession number MK578253-578260).
