# Supplementary material for: Temporal escalation of Pyrethroid Resistance in the major malaria vector Anopheles coluzzii from Sahelo-Sudanian Region of northern Nigeria
Source: Sci Rep. 2019 May 14;9:7395. doi: 10.1038/s41598-019-43634-4 (PMC6517445; doi:10.1038/s41598-019-43634-4)
Supplement: Supplementary file 1 — Supplementary Information [file 41598_2019_43634_MOESM1_ESM.pdf]

Temporal escalation of Pyrethroid Resistance in the major malaria vector *Anopheles coluzzii* from Sahelo-Sudanian Region of northern Nigeria

Sulaiman S. Ibrahim[1,2*], Muhammad M. Mukhtar[2], Jamila A. Datti[2], Helen Irving[1], Michael O. Kusimo[3], Williams Tchapga[3], Nura Lawal[4], Fatima I. Sambo[5] and Charles S. Wondji[1,3*]

[1] Vector Biology Department, Liverpool School of Tropical Medicine (LSTM), Pembroke Place, L3 5QA, United Kingdom

[2] Department of Biochemistry, Bayero University, PMB 3011, Kano, Nigeria

[3] LSTM Research Unit, Centre for Research in Infectious Diseases (CRID), P.O. Box 13591, Yaounde', Cameroon

[4] Department of Biochemistry and Molecular Biology, Federal University Dutsinma, PMB 5001, Katsina, Nigeria

[5] Department of Biological Sciences, Yusuf Maitama Sule University, PMB 3220, Kano, Nigeria

*Correspondence: SulaimanSadi.Ibrahim@lstmed.ac.uk; Charles.Wondji@lstmed.ac.uk; Tel: +44-74-4044-3871

Authors email addresses

Sulaiman S. Ibrahim: SulaimanSadi.Ibrahim@lstmed.ac.uk

Muhammad M. Mukhtar: muhammadmahemukhtar@gmail.com

Jamila A. Datti: jamila.abudatti87@gmail.com

Helen Irving: Helen.Irving@lstmed.ac.uk

Michael O. Kusimo: gkusimo@gmail.com

William Tchapga: williams.tchapga@crid-cam.net

Nura Lawal: nlbatagarawa@gmail.com

Fatima I. Sambo: fatimasambo23@gmail.com

Charles S. Wondji: Charles.Wondji@lstmed.ac.uk

Supplementary Table S1: Knockdown profiles of *An. coluzzii* from Hadiyau, Batagrawa and Ladanai, exposed to permethrin, deltamethrin and DDT

	0 min	15 min	30 min	45 min	60 min
**Had_Permethrin**	0±0.00	1.03±0.24	2.11±0.97	4.2±0.98	5.1±1.16
**Bat_Permethrin**	0±0.00	1.02±0.50	3.29±0.58	6.12±0.60	12.59±0.95
**Lad_Permethrin**	0±0.00	2.1±0.51	6.59±0.58	6.59±0.58	13.26±1.4
**Had_Deltamethrin**	0±0.00	1.11±0.37	2.05±0.96	3.16±1.11	4.4±1.88
**Bat_Deltamethrin**	0±0.00	1.02±0.50	3.06±0.50	5.1±0.55	12.24±1.86
**Lad_Deltamethrin**	0±0.00	1.3±0.50	1.6±0.50	3.07±0.96	5.22±1.25
**Had_DDT**	0±0.00	0±0.00	0±0.00	0±0.00	2.8±0.99
**Bat_DDT**	0±0.00	0±0.00	2.13±0.58	4.26±0.81	8.51±0.89
**Lad_DDT**	0±0.00	0±0.00	0±0.00	1.08±0.50	2.15±0.58
**Had_Cyfluthrin**	0±0.00	4.76±0.82	14.28±0.50	28.57±1.85	38.09±1.5

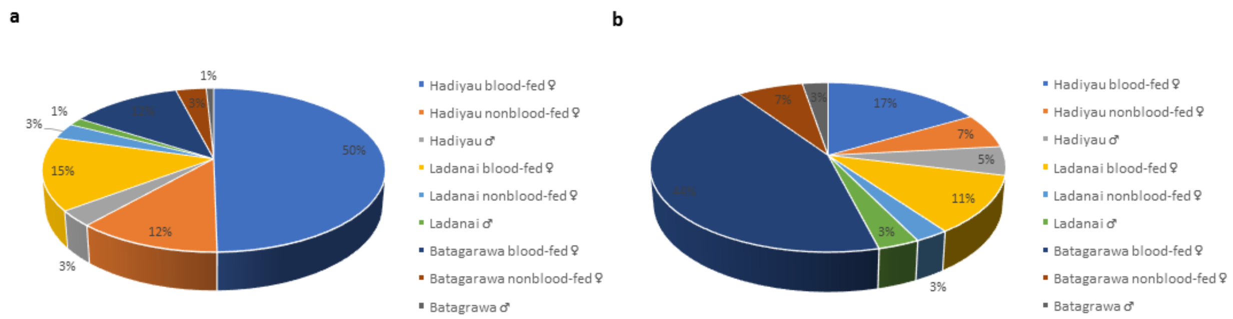

Supplementary Figure S1: Mosquito species distribution and composition in the three sampling sites. (A) *Anopheles coluzzii* and (B) *Culex* species.

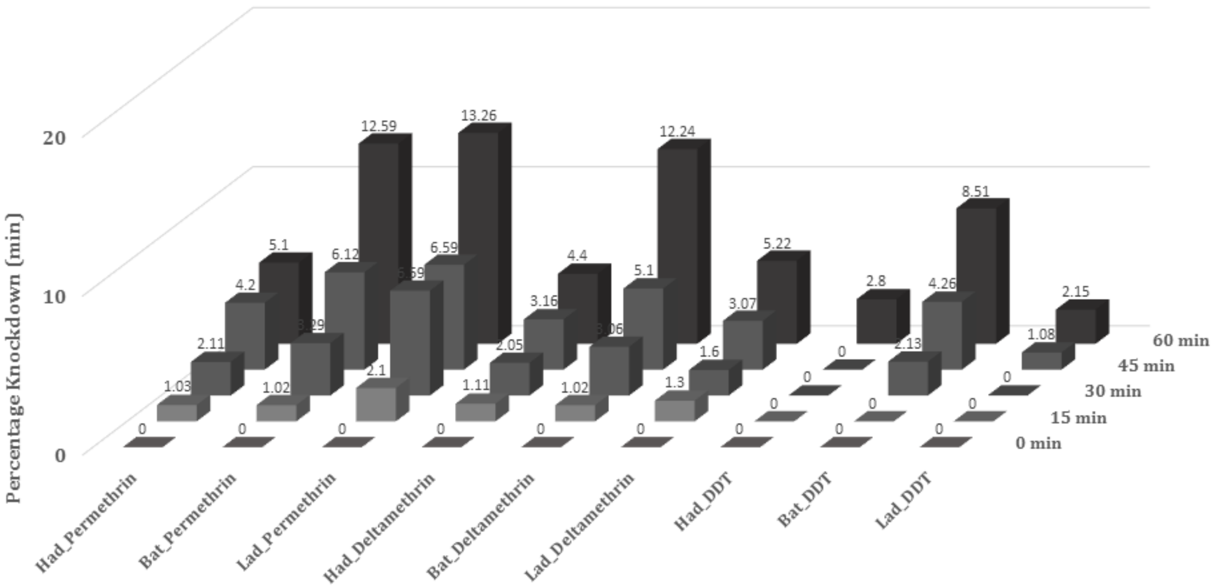

Supplementary Figure S2: 3D plot of knockdown profile of female *An. coluzzii* from Hadiyau (Had_), Batagarawa (Bat_) and Ladanai (Lad_) with permethrin, deltamethrin and DDT. Each bar is a percentage knockdown of values from 4 different replicates from bioassays at different time points.

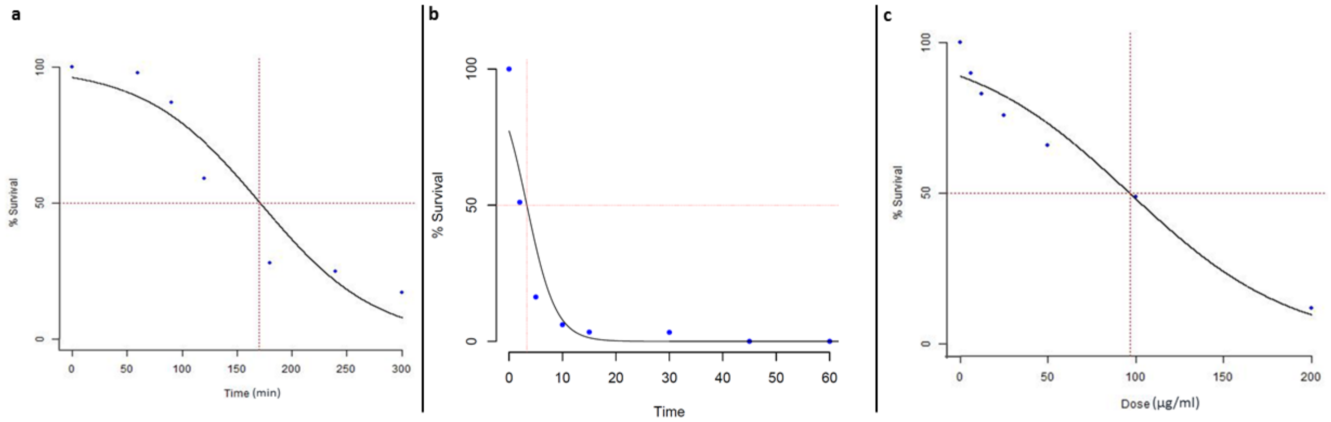

Supplementary Figure S3: Test for strength and frequency of deltamethrin resistance. (A) and (B) Time-course bioassay for LT_{50} estimation for Hadiyau populations and Ngoussou colony with deltamethrin, respectively; (C) dose-response bioassay with Hadiyau populations to establish the LD_{50}.

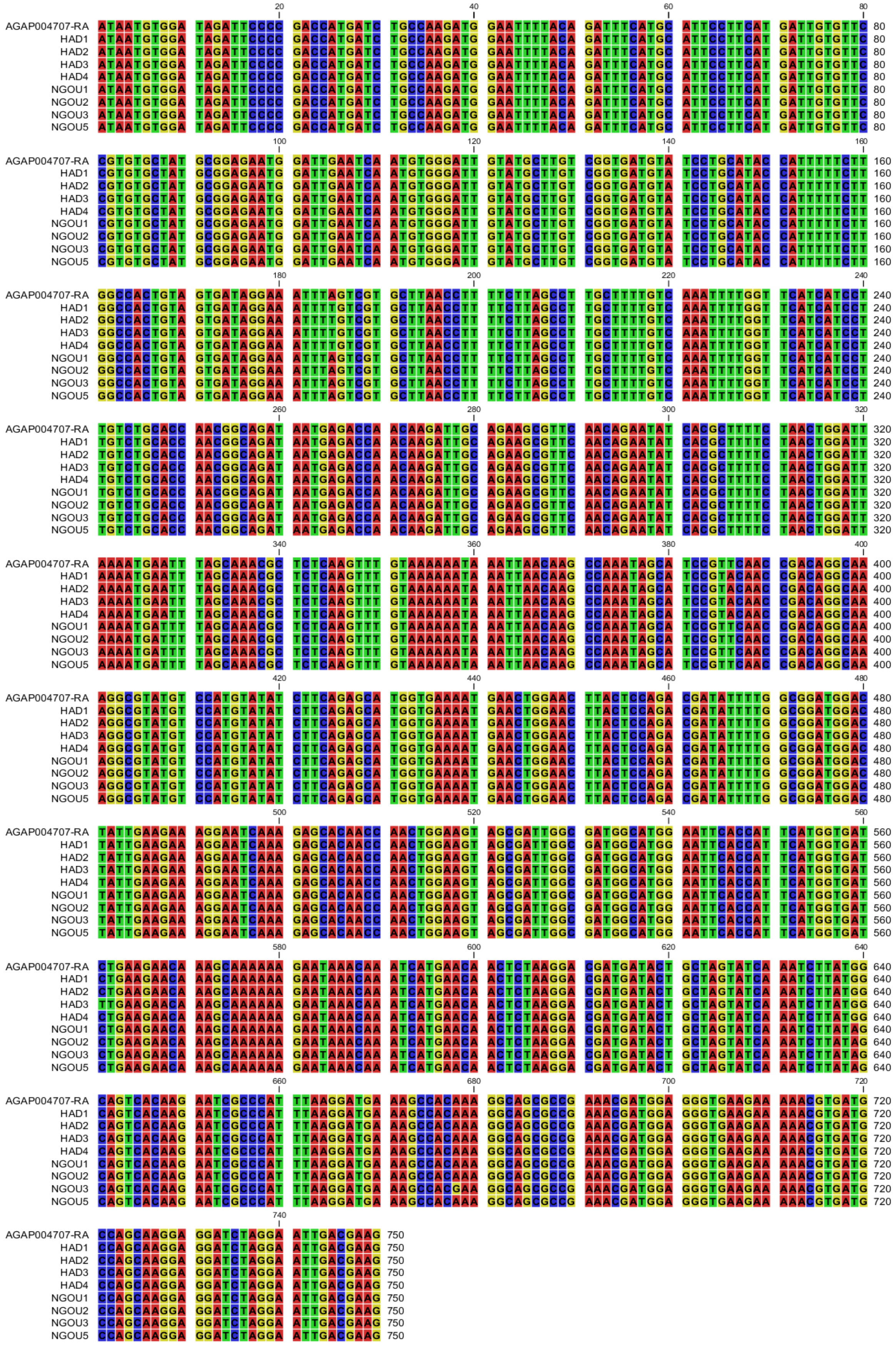

Supplementary Figure S4: Alignment of the exons 18-21 sequences of voltage-gated sodium channel.
